# Clinical management and complications of acute appendicitis in 3 children with SARS-CoV-2 infection: Case report

**DOI:** 10.1097/MD.0000000000040105

**Published:** 2024-10-25

**Authors:** Florin Filip, Monica Terteliu-Baitan, Ramona Avramia, Roxana Filip, Maria Elena Cocuz

**Affiliations:** a College of Medicine and Biological Sciences, Stefan Cel Mare University of Suceava, Suceava, Romania; b Suceava Emergency County Hospital, Suceava, Romania; c Fundamental Prophylactic and Clinical Disciplines Department, Faculty of Medicine, Transilvania University of Brasov, Brașov, Romania; d Clinical Infectious Diseases Hospital of Brasov, Brasov, Romania.

**Keywords:** appendectomy, cecal fistula, children, COVID-19, postoperative abscess

## Abstract

**Rationale:**

Sporadic cases of acute appendicitis (AA) in children with SARS-CoV-2 infection were still recorded at the end of COVID-19 pandemics. We consider that analyses of clinical courses and outcomes is useful to improve the clinical management of such cases in the setting of a general hospital.

**Patient concerns:**

Patient #1 was a 14-year-old girl who presented with nausea, right lower quadrant (RLQ) pain, myalgia, ad low-grade fever for 24 hours. Patient #2 was a 7-year-old boy with a 3-day history of abdominal pain, nausea and vomiting, and fever lasting for 4 days. Patient # 3 was a 16-year-old girl RLQ pain, nausea and vomiting, and fever lasting for 7 days.

**Diagnoses:**

The patients were diagnosed with acute appendicitis (AA) based on the clinical picture, labs and abdominal ultrasound (US) findings. SARS-CoV- 2 infection was diagnosed using rapid antigen test performed at admission.

**Interventions:**

The patients were started on i.v. Ceftriaxone and Metronidazole, antalgics and *i.v*. fluids at admission. Appendectomy was performed the day after admission in patients # 1 and #2, and after 48 hours in patient #3.

**Outcomes:**

Patient #1 had no complications and was discharged on postoperative day (POD) #5. Patient #2 developed a cecal fistula on POD #4 which was treated conservatively with Ertapenem, *i.v*. fluids, and local placement of colostomy bag. The fistula closed spontaneously on POD #12. He was discharged on POD #17. Patient #3 developed a postoperative abscess on POD # 6 and required laparoscopic surgical drainage of the abscess. She was discharged after another 6 days (POD #12). No patient required ICU admission, steroids, or supplemental O_2_ use during their hospitalization. There were no late complications or readmissions in these patients.

**Lessons:**

We consider that AA in these SARS-CoV-2 positive children had a similar course with SARS-CoV-2 negative cases. Compliance to previously established COVID-19 protocols was useful to improve the outcome. The parents should bring the sick child early to the hospital in order to avoid complications related to delayed presentation and not to SARS-CoV-2 infection itself.

## 1. Introduction

The impact of the COVID-19 pandemic on health systems worldwide, particularly on surgical specialties cases, has been thoroughly presented in the literature.^[1–4]^ Compared to the adult population, children had a lower incidence of infection and severe disease, possibly due to a lower expression of angiotensin-converting enzyme-2 (ACE-2) receptors within the nasal passages and, subsequently, fewer viral binding sites.^[5,6]^ Early cases diagnosed in China showed that most children were asymptomatic or had only mild to moderate symptoms. Children with severe manifestations presented with gastrointestinal involvement (pain, vomiting, or diarrhea), mimicking acute abdomen (including acute appendicitis [AA]) in many cases.^[7–9]^ The association between COVID-19 and AA in children was largely debated, as gastrointestinal symptoms in SARS-CoV-2 infection can cover or mimic appendicitis.^[10–12]^ Some research data outlined a possible involvement of SARS-CoV-2 infection in the etiology of AA, explained by the large number of ACE-2 receptors in the terminal ileum. It has been postulated that terminal ileitis and/or lymphoid hyperplasia associated with COVID-19 could represent the starting point for AA.^[13,14]^

Many articles pointed out that the COVID-19 pandemic limited access to specialized care in many areas, especially in low- or middle-income countries. This issue could have delayed the diagnosis and definitive treatment of acute appendicitis in the pediatric population.^[15,16]^ Some,^[17–19]^ but not all^[20,21]^ studies performed during the early phases of the COVID-19 pandemic suggested that delayed hospital presentation of children with AA was responsible for a higher incidence of complicated cases. As a result, the clinical management of AA cases in children had to be adapted to the new epidemiological conditions to generate an optimal outcome.^[22,23]^ The overall influence of COVID-19 on pediatric surgical activity could have been significant, particularly in developing countries.^[24–26]^ Emergency cases and a significant portion of urgent cases, as per the prior classification, exemplify scenarios where delaying surgical intervention would likely lead to unfavorable out-comes for most patients. Apart from evaluating the risks and benefits for patients, clinicians must also take into account the risk of disease transmission to themselves and the strain on hospital resources. Healthcare professionals, including pediatric surgeons, were regarded as a high-risk group for COVID-19.^[27]^

Although the number of SARS-CoV-2 infections decreased significantly in early 2022, sporadic cases were still recorded.^[28]^ The experience gained during the COVID-19 pandemic was very helpful in such cases. We present our experience with 3 (three) cases of acute appendicitis in SARS-CoV-2 positive patients who were treated in our department after the COVID-19 pandemic was declared ended by the health authorities. Two of them developed significant complications (one peritoneal abscess which required surgery and one fistula which healed with conservative treatment, respectively); the third patient had an uncomplicated course. Appropriate clinical management was offered in the COVID-19 pediatric area of the hospital. The outcome was favora-ble in all 3 cases.

## 2. Materials and methods

The study was conducted in accordance with the Declaration of Helsinki, and approved by the Ethics Committee of Suceava Emergency County Hospital, protocol code 28/29.04.2023.

Several previous studies regarding COVID-19 and pediatric surgical cases^[29–31]^ were used to define the methodology of this article. We reviewed the hospital records of children treated in our department between April 01, 2022, and March 31, 2023. The total number of patients was 984, of which 6 (0.6%) patients were COVID–19–positive and 197 (20.02%) patients were diagnosed with acute appendicitis (AA). Only 3 (1.52% of AA group) patients tested positive for SARS-CoV-2 infection. Patients’ demographics, age, the interval between the onset of symptoms and the initial presentation to the emergency room (ER), and the clinical picture at admission were analyzed. According to the literature, complications in AA cases were represented by abscess, peritonitis, perforated appendicitis, or the occurrence of postoperative fistula. Patients with no local or systemic complications during their admission were classified as non-complicated. Screening for SARS-CoV-2 infection was per-formed at admission using rapid antigen testing, according to the COVID-19 protocol used in our hospital. Laboratory data, ultrasound (US) studies, and postoperative pathology results obtained in these patients were also included in the article. The outcome was represented by discharge without complications.

## 3. Results

The most relevant clinical and laboratory data for these patients are presented below:

**Case 1**: A 14-year-old girl with a 1-day history of nausea, right lower quadrant (RLQ) pain, myalgias, and fever (37.4 °C) presented to the ER where she tested positive for COVID-19 (rapid antigen test). She had no cough, dyspnea, or skin rash. Her vitals at admission were as follows: BP = 105/50 mm Hg, HR = 70/min, RR = 14/min, SpO_2_ = 98% in room air. She had increased white blood cell count (WBC) count with neutrophilia, lymphopenia, and monocytosis: lymphocytes = 12.5% of total WBC (Normal values = 1.30–4.00 × 10^3^ µL/21.00–40.00% of total WBC), neutrophils = 77.7% of total WBC (Normal values = 2.00–7.50 × 10^3^ µL/40.00–75.00% of total WBC), and monocytes = 7.1% of total WBC (Normal values = 0.15–0.70 × 10^3^ µL/3.0–7.0% of total WBC). Blood chemistry was normal: serum creatinine = 0.79 mg/dL (normal values = 0.55–1.02 mg/dL), ALAT = 14 U/L (normal values = 9–24 U/L), blood glucose = 87 mg/dL (normal values = 60–99 mg/dL).

Abdominal US study identified an 11-mm diameter hypoechoic tubular structure located in the RLQ, suggestive of acute appendicitis, and free 5-mm width peritoneal fluid between the ileal segments. She was admitted to the pediatric COVID-19 area and started on *i.v*. fluids, Ceftriaxone and Metronidazole, analgesics. An appendectomy was performed after 12 hrs and the patient was transferred to the COVID-19 surgical area after surgery. On postoperative day #1 (POD #1) she had a fever spike of 39°C, but had no dyspnea and required no O_2_. Her labs at admission: total WBC = 7. 3 × 10^3^ µL with lymphopenia (1.17 × 10^3^ µL/15% of total WBC), normal neutrophils and monocytes; CRP = 2.94 mg/dL (N < 0.5 mg/dL); LDH = 173 U/L (normal values = 157–272 U/L); serum fibrinogen = 351 mg/dL (normal values = 150–390 mg/dL) and D-dimers = 0.51 µg/mL FEU (normal values < 0.5). A chest X-Ray (CXR) showed no changes suggestive for COVID-19. She received the same treatment with *i.v*. hydration, analgesics, Ceftriaxone and Metronidazole, and bed rest. Another febrile episode (39.2°C) was recorded on POD #3, but she again had no respiratory symptoms and the clinical course was finally favorable. Labs (WBC, CRP, D-dimers, LDH, serum fibrinogen) were repeated on POD #5 and were all normal. The patient was discharged on POD #5 with regular instructions for home isolation and treatment with oral supplements (Vitamin C and Isoprinosine).

**Figure 1.**
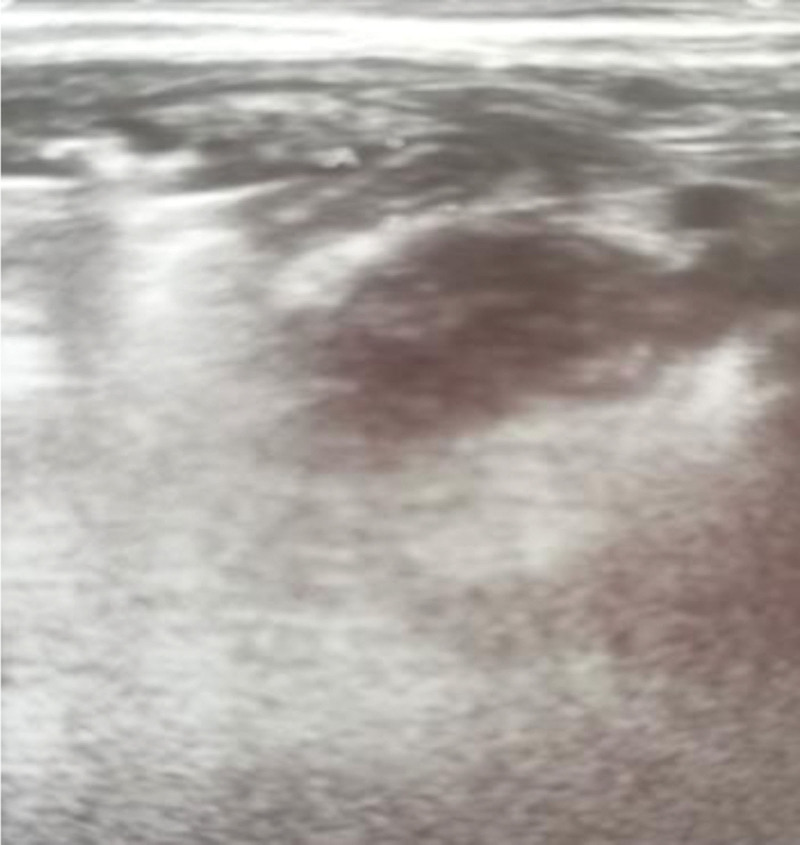
Abdomen ultrasound in patient #1 showing inflamed appendix.

**Case 2**: A 7-year-old boy presented with a 3-day history of abdominal pain, nausea and vomiting, and a fever of 38°C lasting for 4 days. Had no skin rash, cough, or dyspnea. He tested positive for COVID-19 and admission (rapid antigen test). His admission labs were: Ly = 18.7% (N = 1.5–7/20–55%); Mo = 10.4% (N = 0.3–1/<10%); Neu = 70.1% (N = 1.5–8/30–75%); ESR = 72 mm/h (N < 15 mm/h); CRP = 9.8 (N < 0.5) mg/dL. Chest X-ray showed no changes suggestive of COVID-19. The abdominal US identified an 8-mm dilated, painful tubular structure in the RLQ, and 13/8 mm lymphadenopathy; no free peritoneal fluid (Fig. 1). He was started on Ceftriaxone and Metronidazole, *i.v*. fluids, and bed rest. The clinical picture was suggestive of acute abdomen- surgery was performed (appendectomy, lavage, and peritoneal drainage).

During surgery, we identified a gangrenous perforated appendix with localized peritoneal reaction and multiple intestinal adhesions. Peritoneal cultures were taken during surgery (they were positive for *E. coli* and *P. aeruginosa*). He was started on Ertapenem after surgery and returned to the COVID-19 pediatric area. On POD #3 he developed drainage at the level of the incision, which in 24 hours progressed to a cecal fistula with moderate output. A colostomy bag was placed on the incision site to allow spontaneous fistula drain-age. Appropriate *i.v*. fluids were prescribed and Ertapenem was continued. On POD #7 the patient started to present regular bowel movements while the fistula was still draining. The clinical course was favorable and the patient was started on a clear liquid diet. The fistula closed spontaneously on POD #12. The patient was discharged on POD #17 with no complaints. Multiple labs were taken during his admission, which are presented in Table 1.

**Table 1:** Lab data in patient #2.

Date	WBC	Ly, 10^3^/µL/%	Mo, 10^3^/µL/%	Neu, 10^3^/µL/%	ESR, mm/h	CRP, mg/dL	Platelet count, 10^3^/µL	D-dimers	Hb/Ht	LDH U/L
Admission	19.75	3.69/18.7	2.05/10.4	13.86/70.1	72	9.8	–	1.01		
1/26/2023		1.74/17.5			36	6.14	553	6.28	11/32.5	197
1/28/2023		1.77/17.7				4.13	666	3.72		
2/1/2023	-	-	-	-	25	1.15	750	1.99		
2/6/2023	Normal	-	-	-	27	0.35	674	1.01		

CRP = protein reactive C, N < 0.5 mg/dL, ESR = erythrocyte sedimentation rate, normal range < 15, Hb/Ht = hemoglobin/hematocrit, normal range 11.5–14.5 g%/33–41%, LDH = lactate dehydrogenase, normal range = 192–321 U/L, Ly = lymphocytes, normal range 1.5–7/20–55, Mo = monocytes, normal range 0.3–1/<10%, Neu = neutrophils, normal range 1.5–8/30–75%, WBC = white blood cells, normal range 5–14.5.

**Figure 2.**
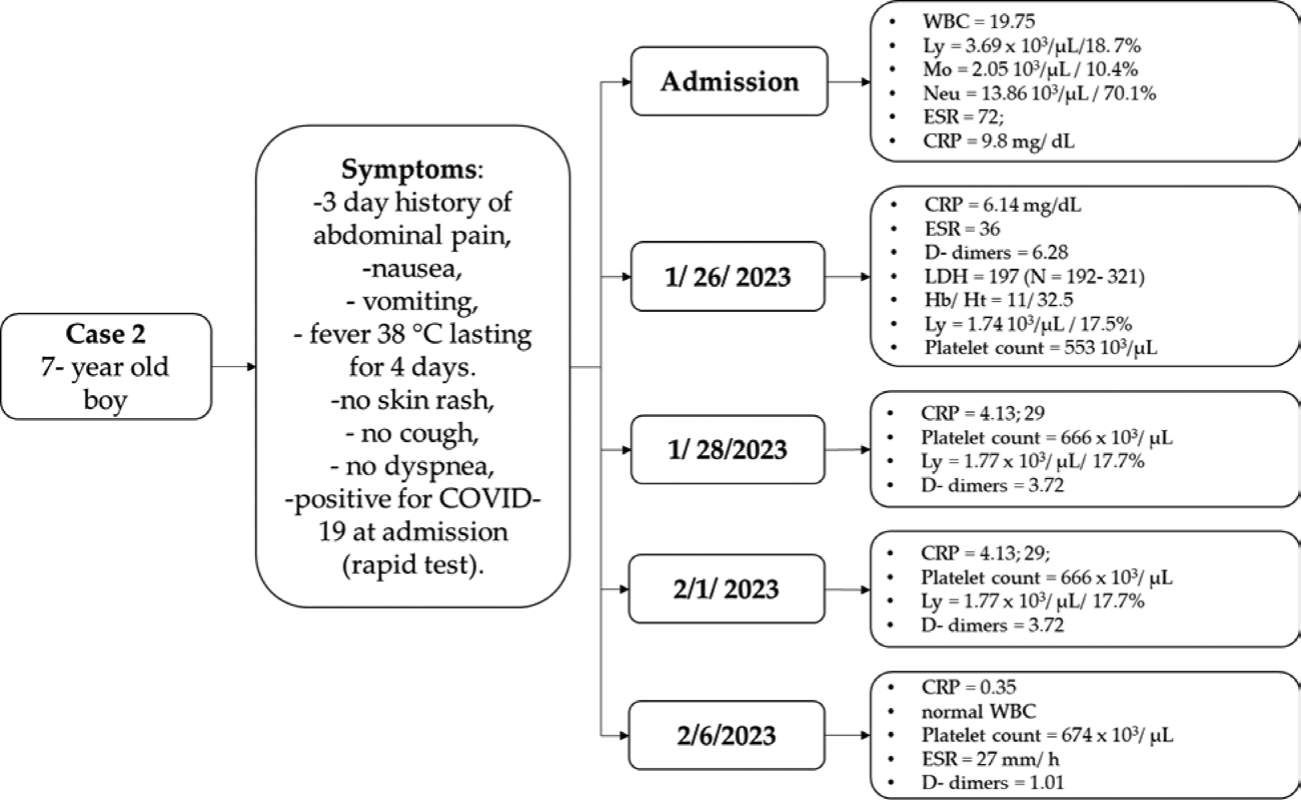
Clinical course of patient #2.

Figure 2 summarizes the clinical course of this complicated case of acute appendicitis.

**Figure 3.**
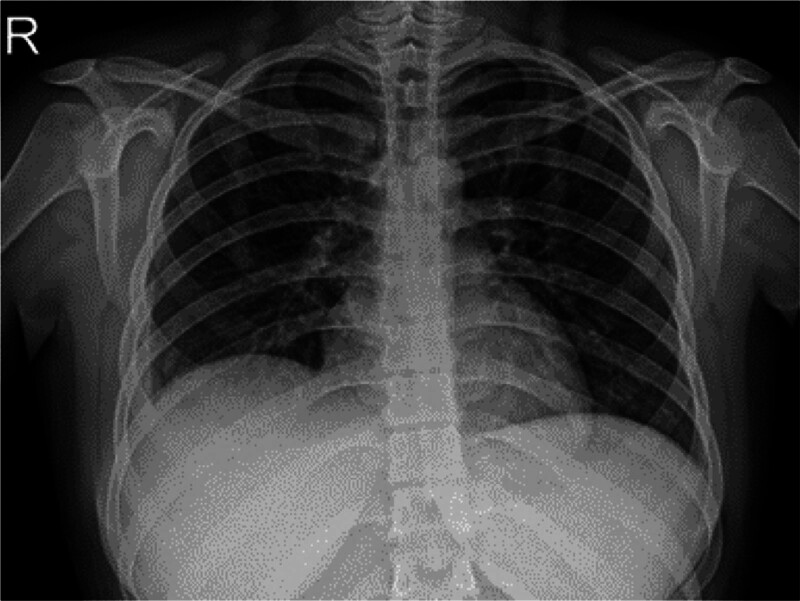
Normal appearance of Chest XRay in patient #3.

**Case 3**: A 16-year-old girl was admitted for RLQ pain, fever of 38°C, nausea, and vomiting lasting for a week. She had received symptomatic treatment with analgesics and Ciprofloxacin at home; her condition worsened and she was referred to our hospital. At admission, her vitals were as follows: BP = 110/60 mm Hg, HR = 82/min, SpO_2_ = 97–98% in room air, RR = 14/min. She had no rash, conjunctivitis, or dyspnea. The abdominal US study showed dilated bowel loops and free peritoneal fluid in moderate amounts between the intestinal loops. A rapid COVID-19 test performed in the ER was positive, so the patient was admitted to the buffer pediatric area and started on analgesics, *i.v*. fluids, Ceftriaxone and Metronidazole *i.v*., bed rest. The abdominal pain persisted over the next 48 hours and she had another 2 episodes of fever up to 38°C. A Chest X-ray showed no pulmonary lesions (Fig. 3).

The WBC was repeated after 48 hours: total WBC = 14.37 × 10^3^/µL with lymphopenia (1.66 × 10^3^/µL/11.6% of total WBC), monocytosis (1.55 × 10^3^/µL/10.8% of total WBC), and increased neutrophils (11.05 × 10^3^/µL/76.8% of total WBC). The CRP level was also increased to 34.04 mg/dL. The clinical picture was highly significant for AA, supported by increasing levels of neutrophils and CRP. An appendectomy was performed that day, which identified a perforated appendix with generalized peritonitis and multiple adhesions. A peritoneal fluid culture was taken during surgery (final result – no bacterial or fungal growth). She was switched on Ertapenem and Metronidazole after surgery and transferred to the COVID-19-positive pediatric ward. However, on POD #6 she developed progressive abdominal pain and a fever of 38°C. The abdominal US identified 2 large residual peritoneal collections located below the liver and in the lower abdomen. The labs were as follows: CRP = 28.31 mg/dL, WBC = 9.03 × 10^3^ with Neu = 6.95 × 10^3^ (77% of WBC) and Mo = 0.71 × 10^3^ (7.9% of total WBC), Hb = 7.2 g/dL, Ht = 22%. She underwent laparoscopic drainage of the 2 collections and was continued on Ertapenem and Metronidazole for another 4 days. The postoperative course was favorable. The labs performed before discharge: WBC = 6.12 × 10^3^; Neu = 3.76 × 10^3^ (61.4% of WBC), Lym = 1.5 × 10^3^ (24.5% of WBC); Mo = 0.72 × 10^3^ (11.8% of WBC); Hb = 7. 9 g/dL; CRP = 11.53 mg/dL. The patient was discharged on postoperative day (POD) #12 (POD #6 after laparoscopy), with proper isolation precautions ac-cording to the COVID-19 protocol in use at that time.

**Figure 4.**
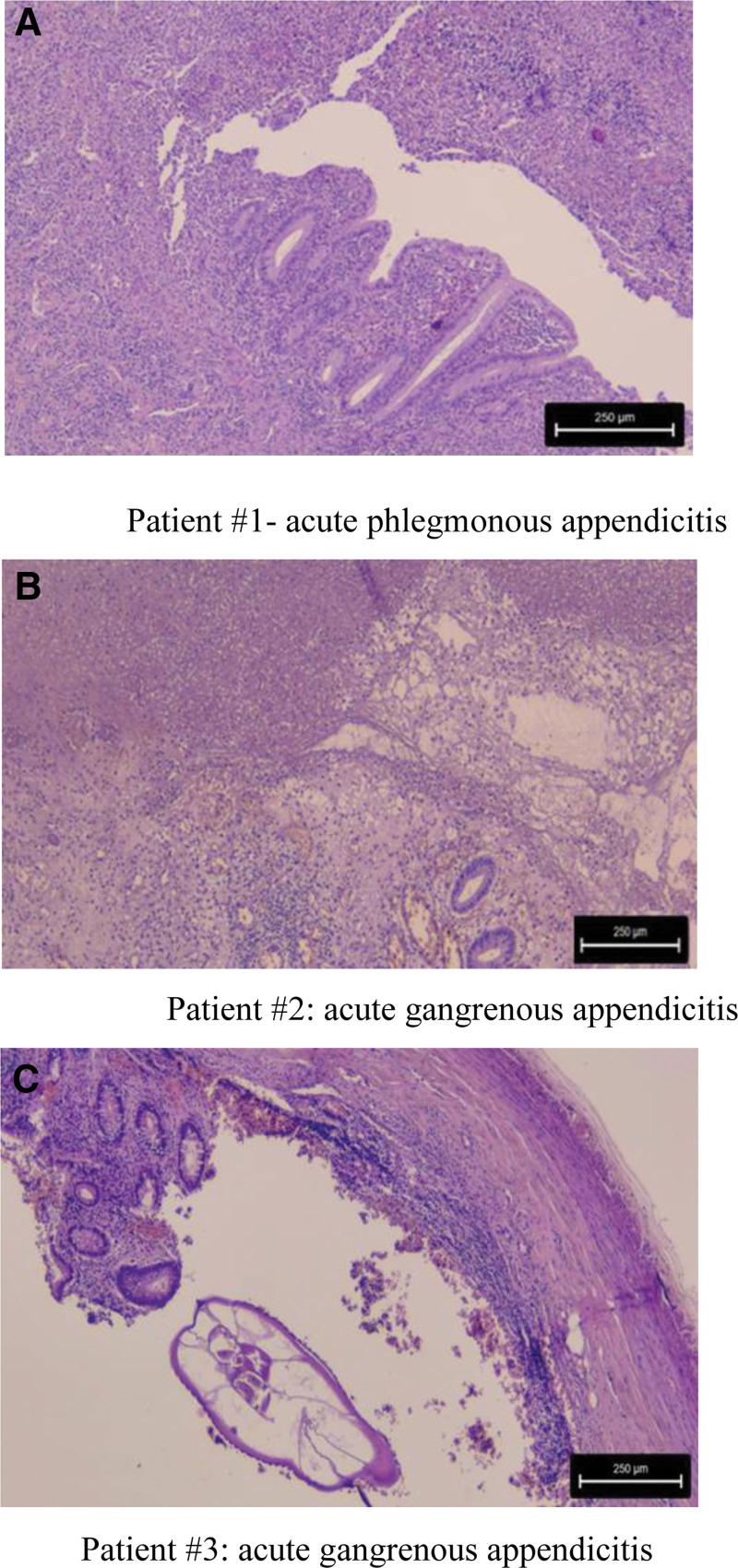
Microscopic appearance of acute appendicitis (Staining with HE – hematoxyllin eosin, Magnification ×200). (A) Patient #1-acute phlegmonous appendicitis. (B) Patient #2: acute gangrenous appendicitis. (C) Patient #3: acute gangrenous appendicitis.

Pathology confirmed the diagnosis of acute appendicitis in all 3 cases. In patient #1 pathology consisted of a discrete polymorphic inflammatory infiltrate of the mucosa; edema, congestion, and focal hemorrhage of the appendiceal wall (Fig. 4A). In patient #2 there was an abundant inflammatory infiltrate with local abscesses in the mucosa; the appendiceal wall presented edema, congestion, and hemorrhage, as well as recent disseminated vascular thromboses. Focal areas of appendiceal wall perforation were also noticed (Fig. 4B). In patient #3, there was a marked inflammatory infiltrate of the mucosa, and congestion of the appendiceal wall. There were also multiple areas of perforation of the appendiceal wall (Fig. 4C).

Table 2 summarizes the main clinical, laboratory, and operative findings obtained from these 3 patients. It is worth mentioning all 3 patients were white-Caucasians and were living in rural areas. None of them had a significant medical history before this episode of acute appendicitis.

**Table 2:** Main characteristics of the COVID-19-positive patients.

No	Gender	Age (years)	History	Admission PE	US Imaging at admission	Operative findings	SARS-CoV-2 Status	Outcome
1	F	14	1-day history of nausea, RLQ pain, myalgia, and fever (37.4°C)	Fever, RLQ pain, stable vitals (BP, HR, RR, and SpO_2_)	11-mm diameter hypoechoic tubular structure located in the RLQ; free 5-mm width peritoneal fluid between the ileal segments	Acute phlegmonous appendicitis	Positive rapid antigen test	Had 2 febrile episodes after surgery; - no Chest Xray findings; - discharged on POD #5 with no complications
2	M	7	4-day history of fever (38°C); 3-day history of RLQ pain, nausea, and vomiting	RLQ pain, stable vitals	8-mm dilated, painful tubular structure in the RLQ; 13/8 mm lymphadenopathy; no free peritoneal fluid	Acute gangrenous perforated appendicitis	Positive rapid antigen test	Developed cecal fistula on POD #4; - resumed regular bowel movements on POD #7; - discharged on POD # 17 with closed cecal fistula
3	F	16	7-day history of intermittent fever, abdominal/RLQ pain, nausea, and vomiting	RLQ pain, nausea and vomiting, stable vitals	Dilated bowel loops; free peritoneal fluid in moderate amounts between the intestinal loops	Acute gangrenous perforated appendicitis, multiple adhesions, peritonitis	Positive rapid antigen test	Developed 2 intraperitoneal abscesses after appendectomy; required laparoscopy for drainage; discharged on POD #12

## 4. Discussion

Acute appendicitis (AA) is one of the most common surgical emergencies in children. It has a lifetime risk between 7% and 9% and a peak incidence in the second decade of life.^[32,33]^ Delayed diagnosis frequently results in escalated disease severity, including increased rates of perforation or intra-abdominal abscesses. Approximately 20% of children with perforated appendicitis are prone to developing post-operative abscesses, a statistic notably contrasting with the mere 0.8% among patients without perforation. Complicated appendicitis has been associated with longer in-hospital admission, increased risk of readmission, and higher financial costs.^[34–36]^ Children, especially those under the age of 5 years, have a higher rate of perforation compared with adults.^[35]^ This risk increases linearly with the duration of symptoms before hospital admission, including children with COVID-19.^[36,37]^ Socioeconomic variables also exert influence on the delayed presentation of pediatric patients, as accessing medical care is contingent upon factors such as parental awareness of the illness, availability of transportation, insurance coverage, and financial standing.^[34,35,38]^

AA represented one of the first surgical diseases to be examined during the COVID-19 pandemic.^[39,40]^ Some papers focused on the impact of COVID-19 on the time of hospital presentation and outcome; it was found that many patients presented late due to lockdown restrictions or policies,^[41–43]^ or the fear of COVID-19 contamination.^[38,44]^ Many hospitals had to change the usual management of AA at the time of limited resources available for these cases during the COVID-19 pandemic.^[45]^ During the onset of the COVID-19 pandemic in 2020, there was a notable decline in presentations at the pediatric emergency department in Italy compared to the corresponding periods in 2018 and 2019. This decrease in hospital visits was primarily due to concerns about contracting SARS-CoV-2. Additionally, patients who did seek medical attention during this peak period experienced more adverse outcomes than those in the control period.^[38,44–48]^ Early during the COVID-19 pandemic, Snapiri et al reported a higher rate of complications in AA patients.^[31]^ A recent study from Toronto, Canada^[49]^ showed the odds of complicated appendicitis increased by 26% for every 1-day increase in symptom duration; the COVID-19 group also had a significantly increased risk of complicated appendicitis or symptom duration.^[18,19]^ Clinical characteristics suggestive of severe disease and worsened outcomes in the COVID-19 group were represented by greater duration of *i.v*. antibiotics, increased surgery length, and a higher proportion of readmissions.^[46,50]^

Recent data allowed a completed analysis of AA rates and severity related to COVID-19. Gerall et al found that AA cases treated in the large New York metropolitan area were characterized by severe clinical course, increased rates of perforation, heightened antibiotic usage, extended hospital stays, and more frequent occurrences of complications.^[38]^ Fisher et al discovered a rise in perforation rates and prolonged symptom duration upon presentation due to restricted healthcare access during the COVID-19 pandemic.^[51]^ A study conducted in Paris, France, observed a rise in pediatric appendicitis cases and longer travel distances during the pandemic^[52]^; nevertheless, there were no significant alterations in outcomes, including length of hospital stay, abscess rate, or readmissions. Similarly, La Pergola found no significant differences regarding the prevalence and the onset of symptoms of AA in children during the peak period of pandemics in early 2020 in Italy.^[46]^ Also, he could not confirm a delay in the presentation of children with AA during pandemics. The CASCADE study in the UK found that the use of diagnostic imaging and non-operative management increased, but outcomes remained relatively un-affected during the COVID-19 pandemic.^[39]^ Two other studies, one from Lithuania and another one from South Africa, found no difference in length of stay, the severity of presentation, or intraoperative findings during the pan-demic in AA patients.^[53,54]^ Similar findings were reported by Tristan et al in their study conducted in Madrid, Spain. The implementation of COVID-19 self-quarantine measures has not led to a rise in the occurrence of complicated appendicitis, and children who did develop this condition did not experience worsened clinical outcomes. Favorable outcomes could be attributed to the re-organization of hospital resources, the implementation of fast-track treatment protocols for non-complicated appendicitis, and the increased utilization of homestay hospitalization for cases of complicated appendicitis. In their series of cases, there was no evidence of parental delay in seeking medical care, nor did emergency department pediatricians overlook the diagnosis of appendicitis.^[55]^ In contrast, Kohler et al stated that throughout the pandemic, notably increased rates of complicated appendicitis were observed, alongside a longer duration from symptom onset to presentation at the emergency unit. However, this extension did not achieve statistical significance for both adults and children. The authors took into account 2 hypotheses: the surge in complicated appendicitis cases could be attributed to a decrease in overall cases, which was driven by reduced instances of uncomplicated appendicitis, while the numbers of complicated appendicitis cases remained steady and other possible explanation could be the elevated proportion of complicated appendicitis cases, necessitating more intricate surgical interventions and a higher frequency of open procedures.^[56]^

Our paper presents 3 cases of acute appendicitis (AA) in COVID-19 children who were treated in our department during the late phase of the pandemic (April 01, 2021 to March 31, 2022). They represented 1.52% of the patients treated for AA during this time interval, reflecting the significantly decreased incidence of COVID-19 cases. The clinical course was similar to AA patients without associated SARS-CoV-2 in terms of hospital presentation, clinical picture, lab data, treatment, and postoperative course. Also, the clinical management was simi-lar to the one previously we have implemented for COVID-19 patients. As mentioned in the literature, we use the initial antibiotic protocol with Ceftriaxone and Metronidazole for both complicated and uncomplicated appendicitis for a minimum duration of 3 days. To be discharged, the patients needed to exhibit afebrile status for at least 24 hours, demonstrate tolerance for a regular diet and achieve adequate pain management with oral medications.^[57]^

Two of our patients developed postoperative complications that are well-known in patients with AA: one post-appendectomy abscess, found in up to 24% of cases, depending on the clinical form and the surgical procedure.^[58,59]^ He underwent redo surgery with drainage of the abscess. The literature data also suggests that a conservative approach could be used in such cases, depending on an individual assessment of the patient^[60–62]^; one post-appendectomy cecal fistula, a rare complication, found in 0.5% of cases, and related to complicated appendicitis.^[63,64]^ This patient was treated conservatively, according to the general recommendation for such cases.^[65,66]^ There has been a lot of discussion in the literature regarding the relationship between SARS-CoV-2 infection and the severity of AA cases in children. The epithelial lining of the appendix displays an increased number of ACE-2 and Furin receptors in children, which allows SARS-CoV-2 to invade the appendix and exert a major damaging effect on the mucosa.^[67,68]^ It is also accepted that ACE-2 is involved in regulating blood pressure and hemodynamics^[69,70]^ which could further amplify the local effect of SARS-CoV-2. In a recent study performed in children with acute appendicitis and COVID-19,^[71]^ it was found that a significant number of these patients presented destructive (phlegmonous, ulcerative, and gangrenous) forms of acute appendicitis. Also, the authors found increased levels of CD3+, CD4+, CD20+, CD68+, CD163+, and CD138 + cells in the appendix, as well as an in-crease in pro-inflammatory cytokines IL-1 and IL-6, and anti-inflammatory cytokines IL-4 and IL-10. There was a predominance of the IL-6/IL-10 ratio, especially in the age group 6 to 12 years, which is considered a predictor of severe forms of severe SARS-CoV-2 infection.^[72,73]^ However, these data are preliminary and need further studies to be confirmed. In our 2 cases who developed complications, a possible explanation for their clinical course was the delayed presentation to the ER (3 and 7 days, respectively) after the onset of symptoms. We do not perform immunohistochemical studies routinely in cases of AA, but it is our impression that none of these complicated cases was specifically related to COVID-19 infection since they had a delayed presentation and no systemic involvement other than AA. The clinical course was similar to non-COVID cases of AA and the outcome was favorable in all 3 cases presented in the article.

There is limited literature regarding the impact of COVID-19 on children with AA in Romania. Costea et al^[74]^ published in 2020 the first case of documented COVID-19-positive child with complicated acute appendicitis diagnosed in Romania. Balanescu et al performed a retrospective study of AA cases treated in a tertiary pediatric hospital in Romania between January 2021 and July 2022; their study aimed to identify clinical laboratory predictors of complicated appendicitis, such as neutrophil-to-lymphocyte ratio and platelet-to-lymphocyte ratio. No data related to changes in the clinical or epidemiological features of AA in children during the SARS-CoV-2-2 pandemics were included.^[75]^ The same authors published in 2022 the results of a survey regarding the management of acute appendicitis in children in Romania which returned 118 answers; however, the impact of SARS-CoV-2 infection in terms of delayed presentation or incidence of complications was not mentioned in their article.^[76]^ Miron et al studied the impact of COVID-19 on emergency presentations and hospital admissions in another tertiary pediatric center from Romania during the first 6 weeks of the pandemic (March–August 2020) compared with the same interval in 2019.^[77]^ They found the number of pediatric emergencies decreased 2.8-fold, but the proportion of major emergencies in-creased significantly (*P* < .001). The number of admissions also decreased 3.3-fold in 2020, compared to 2019, but the risk of admission for respiratory tract infections and respiratory failure increased 1.3- and 2.3-fold, respectively. No special remarks were made regarding the gastro-intestinal emergencies, including cases of AA. Thus, to our knowledge, there are no previous reports of AA cases in COVID-19-positive children from Romania except for the experience presented by our department.

The SARS-CoV-2 infection in our patients was diagnosed using the rapid anti-gen test performed on nasopharyngeal swabs, according to the national proto-col in use at that time. Many assays typically necessitate well-equipped laboratory facilities and skilled personnel. While results are usually obtainable within 2 hours, numerous countries are experiencing delays of up to 7 days. These delays in acquiring molecular testing results can heighten the risk of virus transmission.^[78]^ It was reported that antigen tests can increase the over-all COVID-19 testing capacity; they also have the advantages of shorter turn-around times and lower costs.^[79]^ Antigen tests tend to exhibit optimal performance in individuals with elevated viral loads (Ct values < 25), typically observed during the pre-symptomatic phase (1–3 days before symptom onset) and early symptomatic phase (within the initial 5–7 days of illness) of COVID-19.^[80]^ The benefits of antigen tests, including their affordability and quick results, aid in promptly identifying infectious individuals. The World Health Organization (WHO) recommended rapid tests that could serve as alternatives to laboratory-based real time polymerase chain reaction when immediate patient care decisions are necessary or when real time polymerase chain reaction cannot be provided promptly.^[81]^

This paper has some limitations since it is a single-center, retrospective study. There was no control group. It included a small number of cases treated in our department based on the experience gained during the early phases of the COVID-19 pandemic.^[82,83]^ This approach can be used to introduce and validate universal protocols on resource allocation and proper management for future waves of the COVID-19 pandemic or similar health crises.^[43,49]^ From a teaching perspective, proper management of COVID-19-positive children using available personnel and hospital resources can generate favorable outcomes for these patients. Since the early phase of the COVID-19 pandemic, we have encouraged parents to bring sick children early to the hospital since we can manage properly surgical cases associated with SARS-CoV-2. In our opinion, avoiding delayed presentations is a key factor in preventing the occurrence of complicated surgical cases, including children with AA. The relationship between SARS-CoV-2 infection and the degree of appendiceal involvement is still debatable and de-serves further research to be clarified.

## 5. Study limitations

This article is a single-center, retrospective study, which included a small number of cases. It only included a limited time-interval at the end of the COVID-19 pandemic. No statistical analysis was included. The diagnostic and treatment procedures were performed according to standard procedures in use in the hospital at the time of admission. No regular long-term follow-up visits were scheduled since the patients were instructed to return to the hospital only in case complications would occur.

## 6. Conclusions

We report 3 cases of AA in SARS-CoV-2-positive children who were treated near the end of the COVID-19 pandemic. The clinical course was similar to AA patients without SARS-CoV-2 infection. Two patients developed postoperative complications (1 peritoneal abscess and 1 cecal fistula, respectively), which were treated according to standard protocols. The outcome was represented by the lack of complications or readmission 30 days after discharge. All 3 patients were discharged without complications. No patient required intensive care unit admission, steroids, or supplemental O_2_ use during their hospitalization. The clinical management in these patients was based on COVID-19 protocols already in use in our hospital since 2020. Although the incidence of SARS-CoV-2 infection significantly de-creased after April 2022, sporadic cases were still recorded. Proper management based on the experience gained during the COVID-19 pandemic is very helpful in treating such cases.

## Author contributions

**Conceptualization:** Florin Filip, Ramona Avramia, Roxana Filip, Maria Elena Cocuz.

**Data curation:** Florin Filip, Maria Elena Cocuz.

**Formal analysis:** Florin Filip.

**Investigation:** Florin Filip.

**Methodology:** Maria Elena Cocuz.

**Project administration:** Monica Terteliu-Baitan.

**Resources:** Ramona Avramia.

**Supervision:** Florin Filip, Monica Terteliu-Baitan, Roxana Filip.

**Validation:** Roxana Filip.

**Writing – original draft:** Florin Filip, Monica Terteliu-Baitan, Ramona Avramia, Roxana Filip, Maria Elena Cocuz.

**Writing – review & editing:** Florin Filip, Roxana Filip, Maria Elena Cocuz.
